# Length variations within the *Merle* retrotransposon of canine *PMEL*: correlating genotype with phenotype

**DOI:** 10.1186/s13100-018-0131-6

**Published:** 2018-08-03

**Authors:** Sarah C. Murphy, Jacquelyn M. Evans, Kate L. Tsai, Leigh Anne Clark

**Affiliations:** Department of Genetics & Biochemistry, Clemson University, Clemson, SC 29634 USA

**Keywords:** *SILV*, Dilution, Pigmentation, Dog, SINE, Mononucleotide repeat, Slippage, Exonization, Coat pattern, Alternative splicing

## Abstract

**Background:**

The antisense insertion of a canine short interspersed element (SINEC_Cf) in the pigmentation gene *PMEL* (or *SILV*) causes a coat pattern phenotype in dogs termed merle. Merle is a semi-dominant trait characterized by patches of full pigmentation on a diluted background. The oligo(dT) tract of the *Merle* retrotransposon is long and uninterrupted and is prone to dramatic truncation. Phenotypically wild-type individuals carrying shorter oligo(dT) lengths of the *Merle* allele have been previously described and termed cryptic merles. Two additional coat patterns, dilute merle (uniform, steely-grey coat) and harlequin merle (white background with black patches), also appear in breeds segregating the *Merle* allele.

**Results:**

Sequencing of all *PMEL* exons in a dilute and a harlequin merle reveals that variation exists solely within the oligo(dT) tract of the SINEC_Cf insertion. In fragment analyses from 259 dogs heterozygous for *Merle*, we observed a spectrum of oligo(dT) lengths spanning 25 to 105 base pairs (bp), with ranges that correspond to the four varieties of the merle phenotype: cryptic (25–55 bp), dilute (66–74 bp), standard (78–86 bp), and harlequin (81–105 bp). Somatic contractions of the oligo(dT) were observed in 43% of standard and 51% of harlequin merle dogs. A small proportion (4.6%) of the study cohort inherited de novo contractions or expansions of the *Merle* allele that resulted in dilute or harlequin coat patterns, respectively.

**Conclusions:**

The phenotypic consequence of the *Merle* SINE insertion directly depends upon oligo(dT) length. In transcription, we propose that the use of an alternative splice site increases with oligo(dT) length, resulting in insufficient PMEL and a pigment dilution spectrum, from dark grey to complete hypopigmentation. We further propose that during replication, contractions and expansions increase in frequency with oligo(dT) length, causing coat variegation (somatic events in melanocytes) and the spontaneous appearance of varieties of the merle phenotype (germline events).

**Electronic supplementary material:**

The online version of this article (10.1186/s13100-018-0131-6) contains supplementary material, which is available to authorized users.

## Background

Domesticated dogs (*Canis lupus familiaris*) present with a unique pattern of pigmentation termed merle. Merle coats have two features: 1) a light, diluted base color and 2) random patches of fully pigmented fur. Together, these two characteristics constitute the standard merle phenotype. In some breeds, this phenotype is highly desirable because each dog possesses a unique combination of pigment intensity and spot distribution. A merle coat results from heterozygosity for the semi-dominant *Merle* allele. Homozygosity for *Merle* causes severe hypopigmentation with patches of merling and is associated with visual and auditory defects [1, 2].

**Fig. 1:**
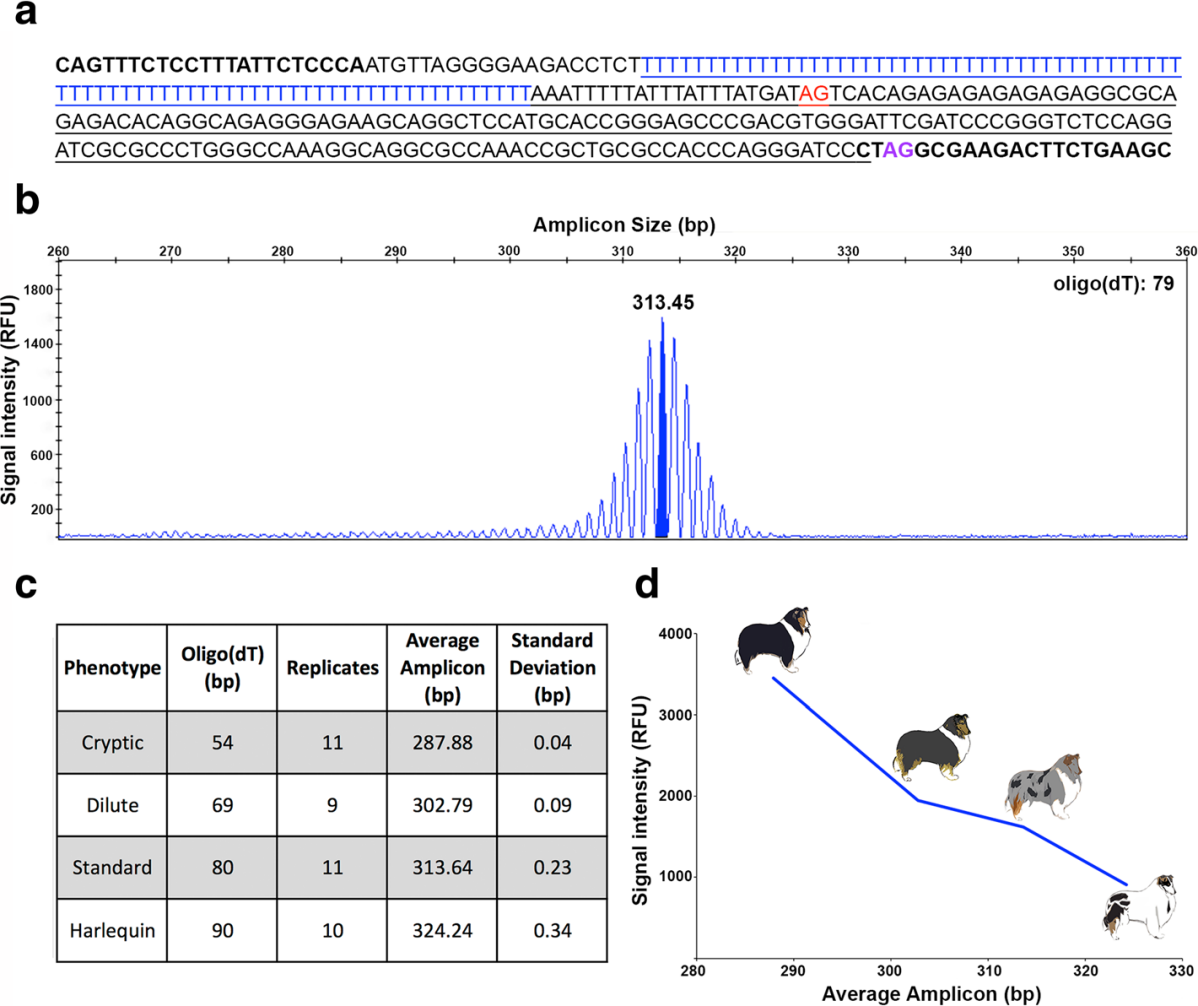
Evaluation of an assay for determining the length of the *Merle* SINE oligo(dT). (**a**) Sequence of the PCR product is shown, with primer sequences in bold. The retrotransposon is underlined, and the oligo(dT) is in blue. Non-oligo(dT) sequences total 234 bp. The wild-type splice site is in purple, while the alternative splice site is in red. (**b**) A chromatogram from fragment analysis depicts amplicon size in base pairs (x-axis) and signal intensity in relative fluorescent units (RFU) (y-axis). For determination of oligo(dT) length, 234 is subtracted from the size of the amplicon peak with the highest RFU (highlighted), rounded to the nearest whole number. (**c**) Standard deviation of amplicon size is given for technical replicates from one dog representing each of four phenotypes. (**d**) Average signal intensity from the technical replicates is plotted against average amplicon size

An early study of merle inheritance revealed a high rate of germinal reversions (3%), leading to the hypothesis that *Merle* is due to a transposable element [3]. Molecular studies later showed that *Merle* is an allele of *PMEL*, also known as *SILV*, a pigmentation gene expressed almost exclusively in melanocytes [4]. This allele contains a canine-specific short interspersed element (SINEC-Cf) inserted at the final intron-exon boundary [5]. The SINE is in an antisense orientation relative to *PMEL*; therefore, its oligo(dA) tail leads the SINE as an oligo(dT) (see Fig. 1a). This orientation creates a pyrimidine-rich tract and an alternative splice acceptor site [6, 7]. In *Merle* dogs, use of the alternative splice site leads to the incorporation of 162 bp of the SINE and some of intron 10 between exons 10 and 11 of the transcript [8]. The reading frame is maintained, resulting in a PMEL protein with a 52 amino acid (aa) insertion [8].

The oligo(dT) tail of the *Merle* retrotransposon is longer and purer relative to other SINEC_Cf’s and is prone to dramatic truncation [8]. Dogs with normally pigmented coats that harbor the retrotransposon insertion, termed cryptic merles, were determined to have an oligo(dT) that is 30 to 40% shorter than standard merles, presumably accounting for the germinal reversions observed by Sponenberg (1984) in breeding studies [3, 5]. This smaller overall insertion size permits use of the original splice acceptor site, allowing for normal production of *PMEL* transcripts and thus functional protein.

Unique to cells producing eumelanin (black or brown pigment), PMEL protein aggregates into fibrillar cross-β-sheets (non-toxic, functional amyloids) in melanosomes, conferring the organelle’s ellipsoid shape and facilitating melanin synthesis [9]. Upon production in the endoplasmic reticulum, PMEL is trafficked to the melanosome where amyloidogenesis takes place [9]. The fibril matrix is essential for full pigmentation, and several hypopigmentation phenotypes of domesticated species have been attributed to mutation of *PMEL*, with many impacting melanocyte viability [10–14].

A modification of heterozygous merles yields harlequin, a pattern of predominantly black patches on a white background. Harlequin in the Great Dane breed is caused by heterozygosity for a dominant-negative mutation in *PSMB7*, encoding an essential subunit of the proteasome [15]. This *PSMB7* mutation is private to Great Danes and inherited independently of *Merle*. The white fur of harlequin Great Danes is attributed to melanocyte death, likely resulting from failure of the impaired proteasome to degrade aberrant PMEL produced during development [15]. Unrelated to the merle phenotype is a uniform dilute coat caused by recessive mutations in *MLPH* that occur in several dog breeds [16, 17].

Dilute and harlequin phenotypes have been reported to appear spontaneously in breeds having *Merle* but not the identified *MLPH* or *PSMB7* alleles. Upon observing that dilute and harlequin phenotypes segregated with the *Merle* allele of *PMEL*, we hypothesized that they may be allelic to *Merle*, rather than caused by independent loci. Here, we investigated *PMEL* in a large cohort of standard and atypical merle dogs. We developed a polymerase chain reaction (PCR) assay for reliable determination of the retrotransposon insertion size and show that the phenotypic spectrum of the merle coat correlates with oligo(dT) length.

## Methods

### Study population

Photos and buccal cells or whole blood were submitted from dogs having merle patterning or presumed to possess the *Merle* allele of *PMEL*. Genomic DNA was isolated using the Gentra Puregene DNA Isolation kit (Qiagen). DNA concentration was quantitated by a NanoDrop 1000 spectrophotometer (Thermo Scientific), and samples were diluted to a concentration of 50 ng/μL.

### Genotyping

Exon 11 of *PMEL* (*M* locus) was initially amplified for each dog using primers described previously [5]. PCR was carried out following manufacturer’s recommendations for Phire Green Hot Start II DNA Polymerase (Thermo Scientific). The following amplification specifications were used: initial denaturation at 98 °C for 3 min; 9 touchdown cycles of 98 °C for 5 s, 55 °C for 5 s, 72 °C for 15 s, reducing by 0.5 °C each cycle; 26 cycles of 98 °C for 5 s, 52 °C for 5 s, 72 °C for 15 s; and a final extension of 72 °C for 1 min. PCR products were run on a 2% agarose gel to determine *PMEL* genotype. All non-merle and homozygous *Merle* dogs were eliminated from further study.

Dogs that had uniformly dilute coats were genotyped for *MLPH* (*D* locus) by a commercial testing company (VetGen). Dogs with classic harlequin coat patterns were genotyped for *PSMB7* (*H* locus), as described previously [15]. Predominantly white dogs were genotyped for *MITF* (*S* locus), which is another cause of white background fur in dogs, termed piebald. We used the primer pair 5′-GGGTGGTTGAAGACCAGAAA-3′ and 5′-CCGGAAGATGCTGGAGTAAG-3′ to detect the upstream SINE insertion associated with piebald on a 2% agarose gel [18, 19].

### *PMEL* sequencing

All exons of *PMEL* were Sanger sequenced for a harlequin merle Collie, including the SINE insertion at the intron10/exon11 boundary, as described previously [5]. Products were resolved on an ABI 3730xl Genetic Analyzer (Applied Biosystems). Whole genome resequencing data were generated from a dilute merle Collie (SRA: SRS1930674; [20]).

### Oligo(dT) length determination

We designed a PCR for fragment analysis using a 6-FAM-labeled forward primer. Primer sequences are shown in Fig. 1a. PCR was carried out following manufacturer’s recommendations for Phire Green Hot Start II DNA Polymerase. The following thermal cycling parameters were used: initial denaturation at 95 °C for 5 min; 5 cycles of 95 °C for 30 s, 56 °C for 15 s, and 72 °C for 10 s; and 30 cycles of 95 °C for 20 s, 54 °C for 15 s, and 72 °C for 10 s; with a final extension of 72 °C for 10 min. Fragment analysis was performed using capillary electrophoresis on an ABI 3730xl Genetic Analyzer. Data were visualized in GeneMapper (Thermo Fisher Scientific) and calibrated to the 500 LIZ® size standard (Thermo Fisher Scientific). All amplicons with a relative fluorescent unit (RFU) value of 100 or greater were recorded. Length of the oligo(dT) was inferred by subtracting 234, representing all non-oligo(dT) base pairs, from the size of the most abundant amplicon (Fig. 1a & b).

Because of the instability of *Merle*, we sought to test the reproducibility of our method for oligo(dT) length determination. We performed 11 technical replicates for four dogs having diverse oligo(dT) lengths: 54, 69, 80, and 90 bp. Replicates were run across three Eppendorf thermal cyclers. Fragments were subsequently analyzed in multiple runs on one ABI 3730xl Genetic Analyzer. Average amplicon size and standard deviation across the replicates were calculated in Microsoft Excel.

## Results

### Study population

Biological samples were obtained from 345 dogs. We first eliminated dogs homozygous for *Merle* (*n* = 50) and non-merle (*n* = 32) individuals. Of the 19 dogs having uniformly dilute coats, a single Border Collie was determined to be *dd* and omitted from the study. *MITF* genotyping was performed for 65 dogs that had white markings covering at least 50% of their coat; three dogs were determined to be piebald (*s*^*p*^*s*^*p*^) and excluded from further analysis. Eighty dogs possessing traditional harlequin coat patterns were genotyped for *PSMB7*; none were *Hh*.

The final study cohort consisted of 259 purebred dogs (Additional file 1) representing seven breeds: 3 Australian Kelpies, 46 Australian Shepherd Dogs, 23 Border Collies, 4 Cardigan Welsh Corgis, 108 Collies, 5 Miniature American Shepherds, and 70 Shetland Sheepdogs. We then organized dogs into one of five phenotypic categories. Cryptic merles (*n* = 19) had black coats with no evidence of merling. Dilute merles (*n* = 18) had uniform steel-grey coats. Standard merles (*n* = 161) had black or brown dilute coats with patches of full pigmentation. Harlequin merles (*n* = 41) had white coats with patches of full pigmentation. Predominantly solid merles (*n* = 12) displayed nearly fully pigmented coats with minimal evidence of merling. Eight dogs did not have phenotypes consistent with any one of these categories.

### *PMEL* sequences

Sequences representing all coding exons and splice sites of *PMEL* were generated for a dilute merle and a harlequin merle. Other than the oligo(dT) of the SINE, no variants were detected as compared to the reference genome.

### Oligo(dT) lengths

We designed a PCR for fragment analysis using a reverse primer that preferentially amplifies the *Merle* allele by priming off of the SINE insertion. The instability of the *M* allele results in a broader amplicon peak, relative to other types of repetitive sequences (e.g., microsatellites), and resembles a bell curve (Fig. 1b). We used technical replicates (Additional file 2) to calculate the standard deviation between the amplicon sizes for four dogs representing the cryptic, standard, dilute, and harlequin merle phenotypes (Fig. 1c). Standard deviations ranged from 0.04 to 0.34, with longer oligo(dT) lengths showing increasing variability. PCR efficiency, measured by average relative fluorescent unit (RFU) across the replicates, decreased as oligo(dT) length increased (Fig. 1d).

**Fig. 2:**
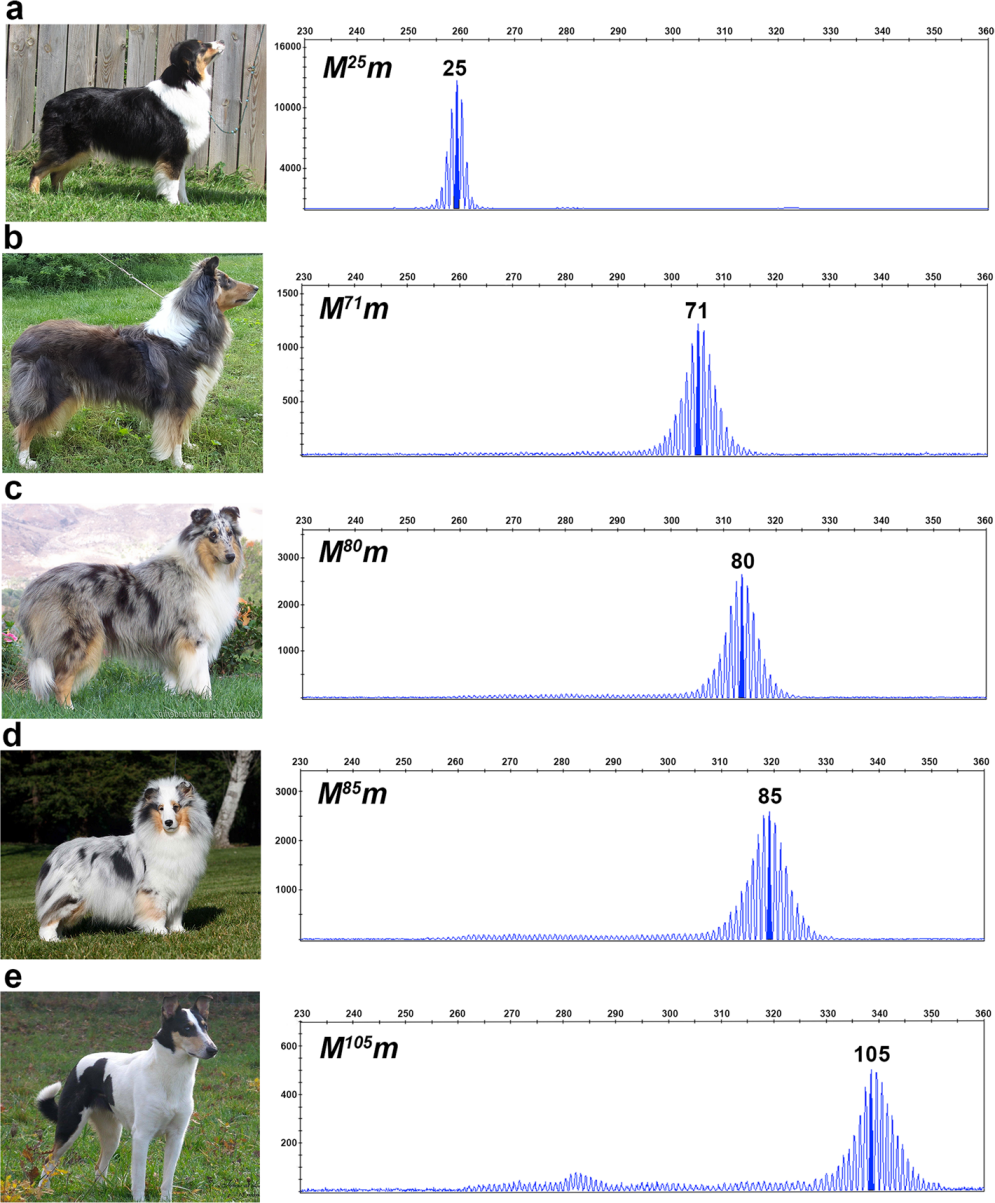
Oligo(dT) lengths correspond to the merle phenotypic spectrum. Photographs and fragment analysis chromatograms are shown for dogs representing each of the four merle varieties: cryptic (**a**), dilute (**b**), standard (**c**), and harlequin (**e**), as well as one dog (**d**) that displays characteristics of both standard and harlequin merles. Amplicon size in base pairs is shown on the x-axis, and the y-axis measures signal intensity (RFU). The most abundant *Merle* amplicon peak is highlighted for each dog with corresponding oligo(dT) length written above. Genotypes are reported in the top left of the chromatogram, with *M* representing the *Merle* allele depicted in the chromatogram and *m* signifying the wild-type allele, confirmed through gel electrophoresis (not pictured). The cryptic and harlequin merle dogs pictured respectively possess the shortest and longest oligo(dT) lengths identified in the study

**Fig. 3:**
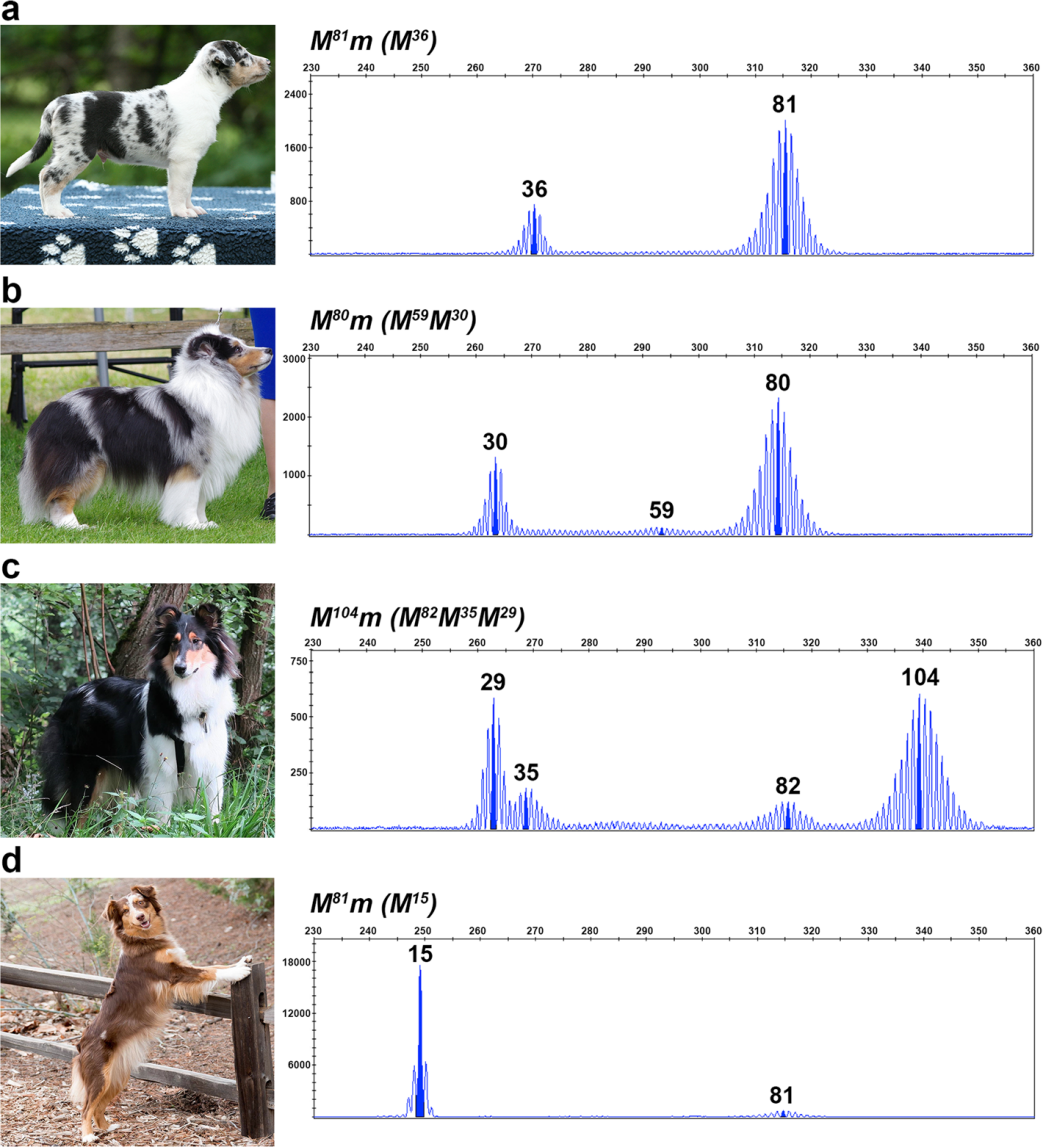
Somatic contractions of the oligo(dT) reflect the proportion of full pigmentation in merle coats. Photographs and chromatograms depicting fragment analysis data from blood and buccal cells are given for two standard (**a** and **b**) and two predominantly solid merle dogs (**c** and **d**). Amplicon size in base pairs is shown on the x-axis, and the y-axis measures signal intensity (RFU). In these four dogs, the amplicon with the longest oligo(dT) represents the inherited allele, while smaller amplicons stem from somatic contractions. Genotypes are listed above each chromatogram, with *M* representing the inherited *Merle* allele, *m* indicating the wild-type allele (not pictured), and *(M)* denoting the contracted *Merle* amplicons

**Fig. 4:**
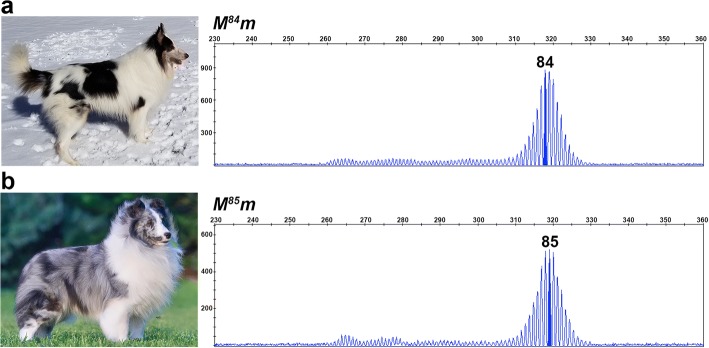
Oligo(dT) lengths of 81–86 bp are observed in standard and harlequin merle dogs. Photographs and chromatograms from fragment analysis data are shown for a harlequin (**a**) and a standard merle (**b**). Amplicon size in base pairs is shown on the x-axis, and the y-axis measures signal intensity (RFU). Genotypes are given for each dog above their chromatogram with *M* denoting their inherited *Merle* allele, and *m* representing their non-pictured, wild-type allele

We detected 36 germline oligo(dT) lengths ranging from 25 to 105 bp (Fig. 2). Cryptic merles (Fig. 2a) had the shortest oligo(dT) lengths, spanning 25 to 55 bp, with an average length of 47.22 bp (*SD* = 9.00). Dilute merles (Fig. 2b) displayed longer oligo(dT) lengths that fell within a smaller window of 66 to 74 bp, with an average length of 70.54 bp (*SD* = 2.39). All standard merles (Fig. 2c) had a primary amplicon (highest RFU), representing an oligo(dT) between 78 and 86 bp, and 43% of dogs possessed one or more additional amplicons with lower RFU values that typically fell within the cryptic range (Fig. 3a and b). The average oligo(dT) length of standard merles was 80.84 bp (*SD* = 1.88). Harlequin merles (Fig. 2e) had oligo(dT) lengths ranging from 81 to 105 bp, and 51% of dogs also possessed one or more smaller amplicons. The average oligo(dT) length of harlequin merles was 87.30 bp (*SD* = 3.96). Standard and harlequin merle dogs having multiple amplicon sizes usually had one or more larger areas of full pigmentation. The standard and harlequin merle ranges overlapped from 81 to 86 bp. Dogs having oligo(dT) lengths within this window displayed either phenotype (Fig. 4). Additionally, seven of the eight dogs that could not be phenotypically classified had oligo(dT) lengths within this window and had coats with characteristics of both merle varieties, as in Fig. 2d.

**Fig. 5:**
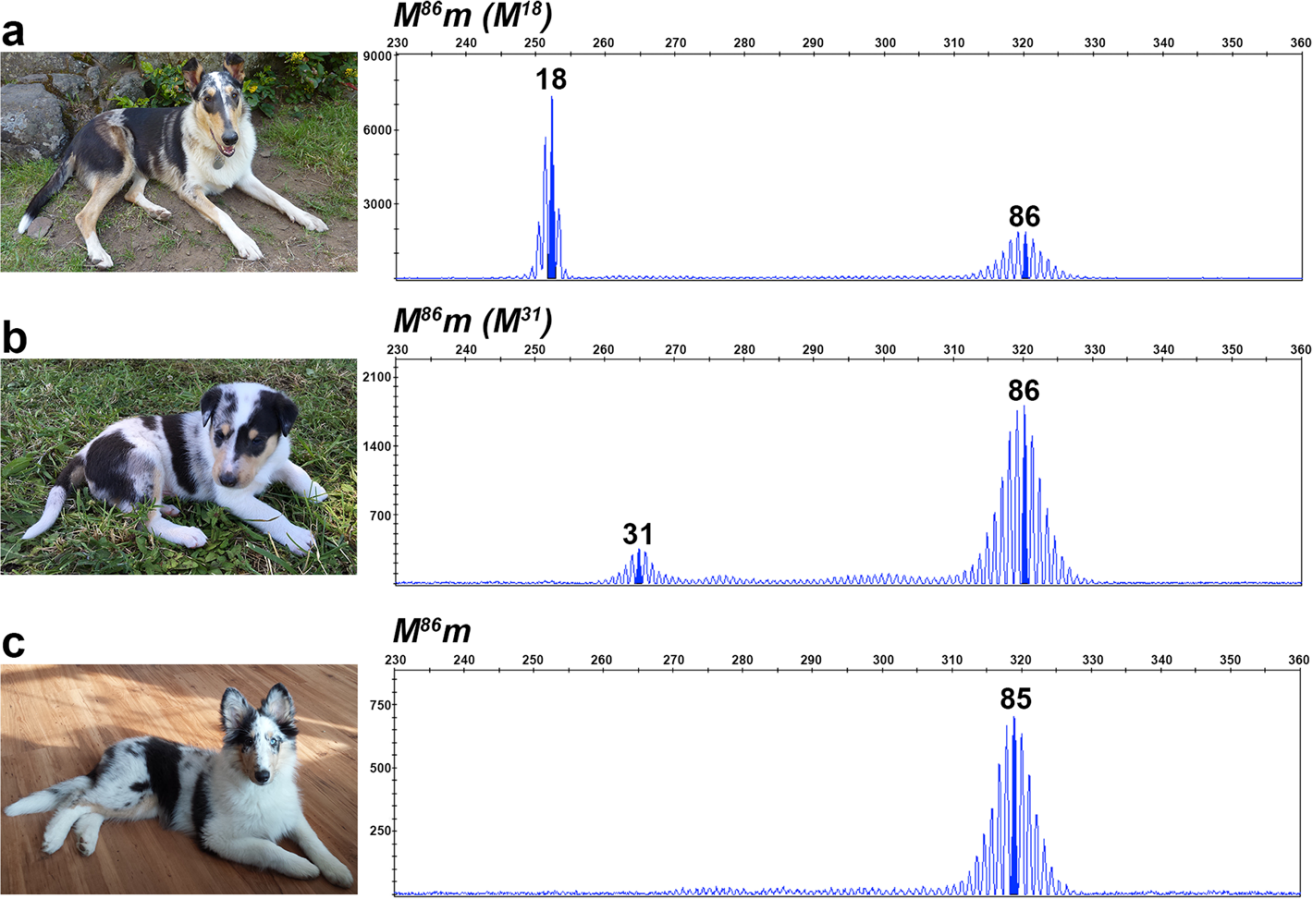
Inheritance of *Merle* oligo(dT) lengths. Fragment analysis chromatograms and photographs are shown for a predominantly solid dam (**a**) and her harlequin merle progeny (**b** and **c**). Amplicon size in base pairs is shown on the x-axis, and the y-axis measures signal intensity (RFU). Genotypes are given for each dog above their chromatogram with *M* denoting the inherited *Merle* allele, *m* representing their non-pictured, wild-type allele, and *(M)* signifying somatic contractions of the inherited *Merle* allele

Dogs having predominantly solid coats and minimal merling had multiple alleles representing varying oligo(dT) lengths, with 58% of dogs having a predominant amplicon in the cryptic range and a minor amplicon of either standard merle (*n* = 2) or harlequin length (*n* = 10) (Fig. 3c and d). A predominantly solid Collie dam with a primary amplicon representing an 18 bp oligo(dT) and a secondary amplicon representing an 86 bp oligo(dT) passed the latter allele to her progeny, indicating that the underrepresented allele exists in the germline (Fig. 5).

**Fig. 6:**
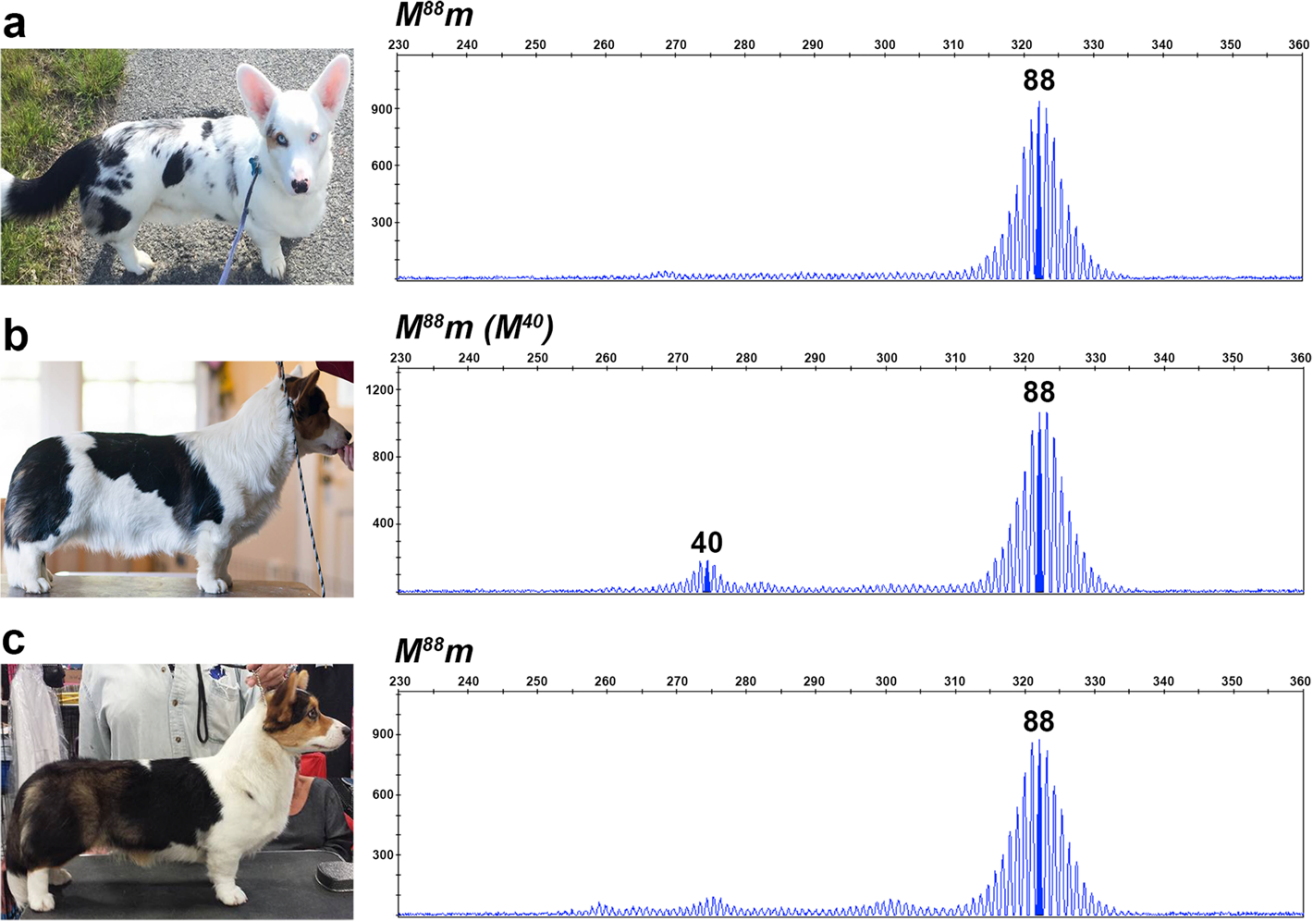
Somatic oligo(dT) contractions and resulting merle phenotypes can vary between individuals. Photographs and fragment analysis chromatograms are shown for three littermates. Amplicon size in base pairs is shown on the x-axis, and the y-axis measures signal intensity (RFU). All three dogs share an identical inherited *Merle* allele in the harlequin range, but possess either zero (**a**), one (**b**), or two (**c**) contracted *Merle* amplicons that correlate with the degree of pigmentation in their coats. Genotypes are listed above each chromatogram, with *M* representing the inherited *Merle* allele, *m* indicating the wild-type allele (not pictured), and *(M)* denoting the contracted *Merle* amplicons

We observed that relatives who inherited the same oligo(dT) length identical by descent often displayed markedly different phenotypes. One example is illustrated in Fig. 6. Three Cardigan Welsh Corgi littermates inherited identical *Merle* alleles from their sire, but display varying amounts of pigment, from typical spotting (Fig. 6a), to moderate patches (Fig. 6b), to large patches (Fig. 6c). The amount of pigmentation present on each dog roughly correlates with the number of cryptic-length amplicons present on their fragment analysis. In a second example, a severely hypopigmented harlequin Collie having the longest observed oligo(dT) length (105 bp; Fig. 2e) transmitted the allele to her daughter (Fig. 3c), who is predominantly solid with multiple cryptic-length amplicons evident in her fragment analysis.

### De novo oligo(dT) lengths

**Fig. 7:**
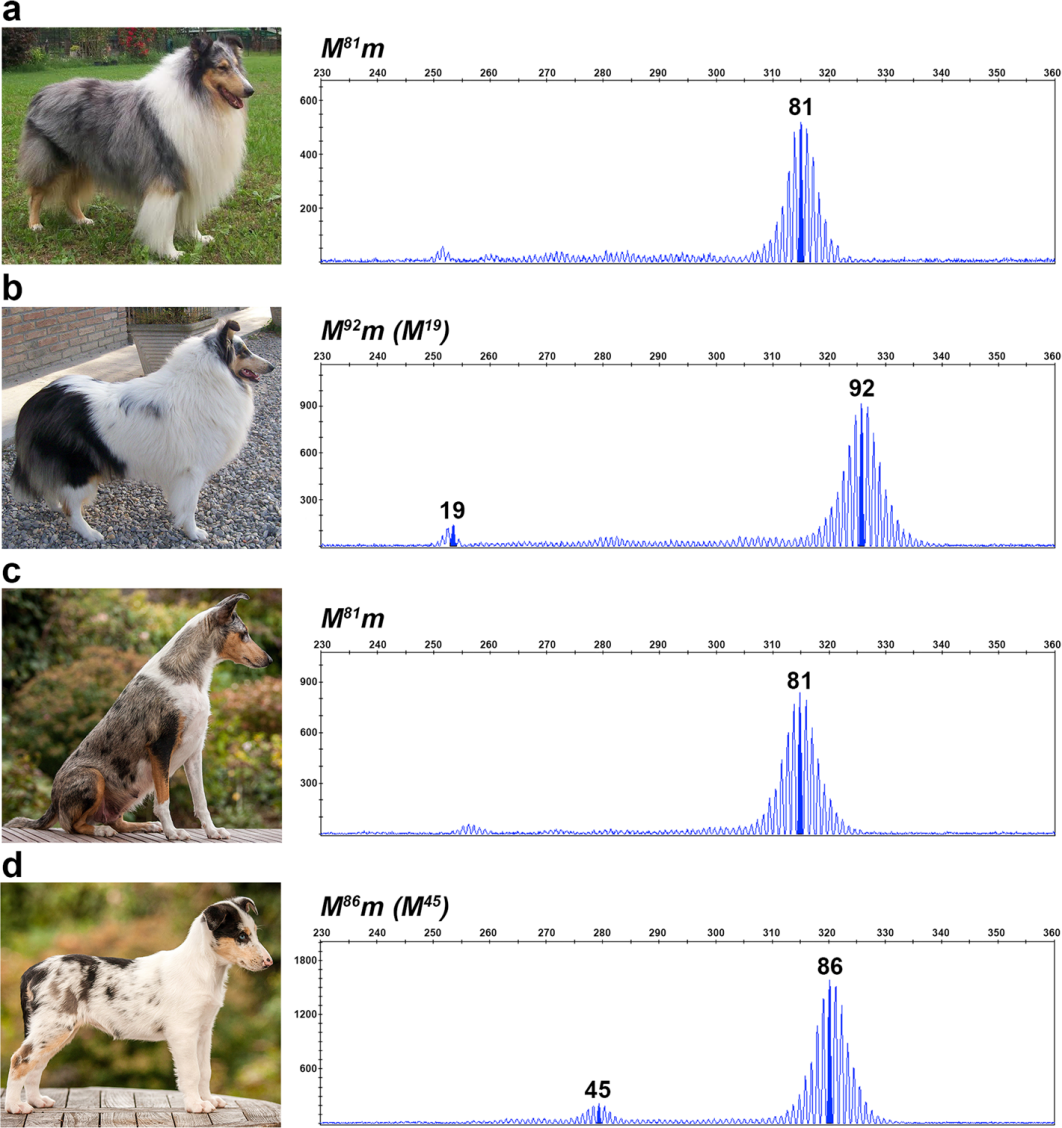
Germline de novo expansion of the *Merle* oligo(dT) tract. Photographs and fragment analysis chromatograms are shown for a standard merle sire (**a**) and his harlequin merle daughter (**b**), as well as a standard merle dam (**c**) and her harlequin merle daughter (**d**). Amplicon size in base pairs is shown on the x-axis, and the y-axis measures signal intensity (RFU). Genotypes are given above the chromatogram for each dog with *M* denoting the inherited *Merle* allele, *m* representing the wild-type allele (not pictured), and *(M)* signifying somatic contractions of the longer de novo *Merle* allele

We observed that seven of the 41 harlequin merles had one standard merle and one non-merle parent (i.e., no harlequin-patterned parent). Their harlequin-length allele can be attributed to de novo expansion of the parent’s *Merle* allele (Fig. 7). In six of the seven incidences, the expanded allele was donated by the sire. We also identified de novo contractions of oligo(dT) length in five of 15 dilute merles for whom we had parental information. A standard merle sire was responsible for contributing the truncated allele in three of the five occurrences.

## Discussion

In this study, we investigated *PMEL* sequences in dogs having variable phenotypes attributed to *Merle* and determined that genetic variation lies exclusively within the oligo(dT) tract of the SINE insertion. In 161 standard merle dogs of different breeds, we found that oligo(dT) lengths fell within a narrow range, with 71% of dogs having tracts of 79, 80, or 81 bp. We use this range as the standard for comparisons of other oligo(dT) lengths herein. Consistent with our previous study [5], the 19 cryptic merle dogs in our cohort had the smallest insertion sizes, with oligo(dT) lengths spanning the bottom 30 bp of the total observed range. We found that two newly described phenotypes, dilute merle and harlequin merle, were also associated with unique oligo(dT) ranges.

### Oligo(dT) length correlates with intensity of background coat color

Harlequin merle dogs harbored the longest oligo(dT) lengths observed and had the most severe dilution phenotype of the merle varieties. This correlation resembles those found in human trinucleotide repeat disorders, wherein expansion of the repetitive sequence is associated with increased disease severity [21, 22]. Other mutations of *PMEL* also result in a complete absence of pigmentation, such as *Dominant white* in chickens and *Silver* in horses [10–13]. These mutations occur within (or immediately following) the transmembrane domain (TMD) of PMEL and cause an abnormally dense clustering of fibrils, resulting in pathologic amyloid and ultimately melanocyte death [13]. Exonization of the *Merle* SINE occurs adjacent to the TMD, suggesting that the white fur of harlequin dogs may result from a similar mechanism whereby the production of abnormal protein negatively impacts melanocyte viability.

Dilute merle dogs, having the darkest background intensity of the dilution phenotypes, possessed oligo(dT) lengths intermediate to those of cryptic and standard merles. The uniform steel-grey coat of dilute merle dogs resembles the Smoky chicken phenotype, which is caused by heterozygosity for a 4 aa deletion in *PMEL* [10]. Haploinsufficiency for PMEL fibrils causes reduced formation of melanin and dark grey plumage, instead of black, in the Smoky chicken [10, 13]. We propose that alternative splicing resulting in SINE exonization and mutant transcripts from the *Merle* allele similarly causes a reduction of PMEL fibers and the steel-grey fur color of dilute merles.

Cryptic merle dogs had the shortest oligo(dT) lengths and no coat dilution. The reduced oligo(dT) tract is seemingly short enough to permit use of the original exon 11 splice site and no alternative splicing occurs. Therefore, the boundary between the oligo(dT) lengths in cryptic and dilute merle dogs should represent the threshold for use of the alternative splice site. In our population, we observed cryptic merle dogs with oligo(dT) lengths up to 55 bp and dilute merles with lengths as short as 66 bp. Thus, we propose that alternative splicing begins to occur with SINE oligo(dT) lengths between 56 and 66 bp.

**Fig. 8:**
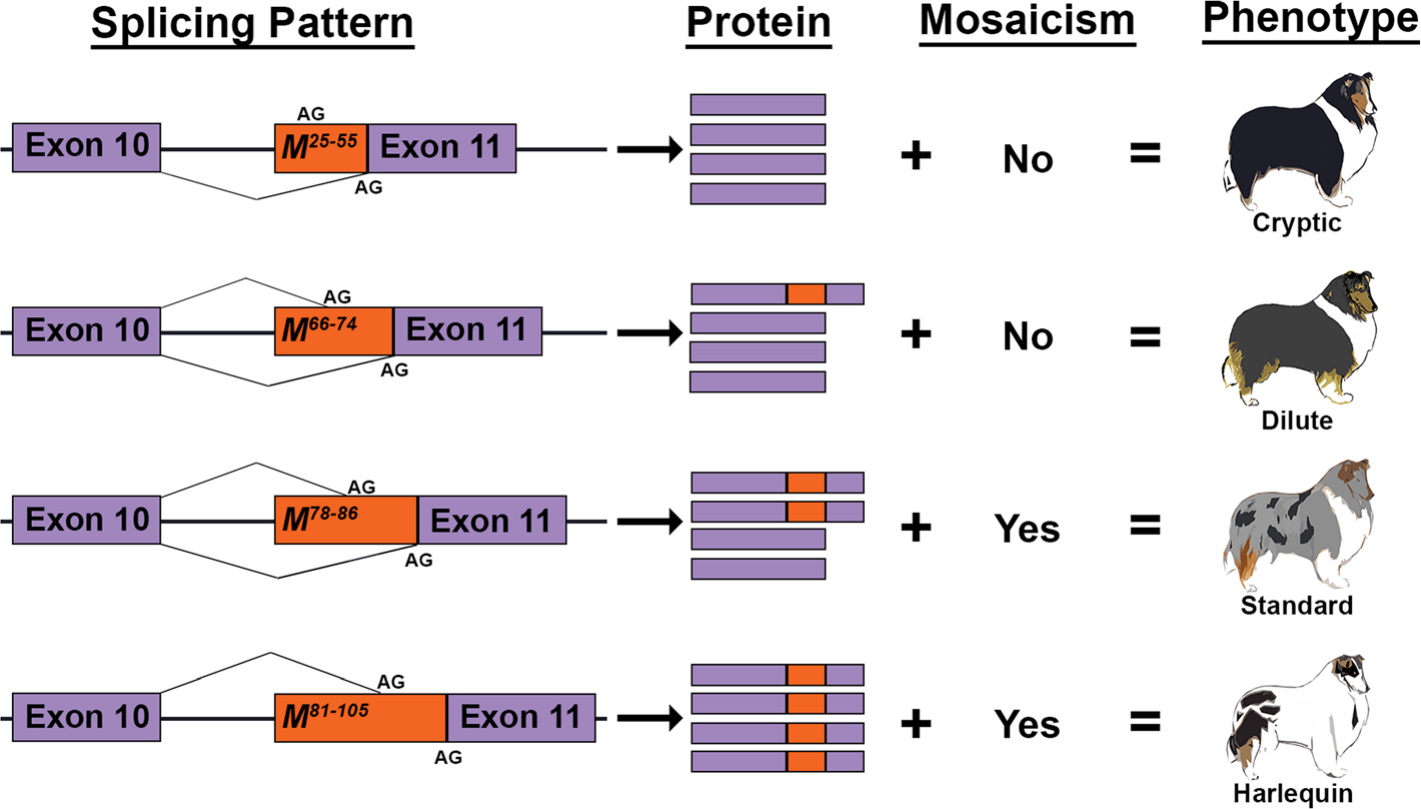
Proposed mechanism for merle phenotypic variation. Suggested patterns of splicing are shown for cryptic, dilute, standard, and harlequin merles. The *Merle* SINE is depicted in orange with oligo(dT) length ranges superscripted. The original and alternative (within SINE) splice acceptor sites are denoted by “AG.” The proportion of wild-type (solid purple) to aberrant (purple and orange) PMEL protein illustrates the proposed frequency of alternative splicing, as it corresponds to oligo(dT) length. Mosaicism reflects whether somatic oligo(dT) contractions were observed in each phenotypic group herein. Together, the rates of alternative splicing and somatic oligo(dT) contractions confer the background coat color intensity and variegation, respectively

Alternative splice sites created by retrotransposon insertions are generally less efficient than the original site, resulting in underrepresentation of alternative transcripts [23]. We propose that the *Merle* allele produces both wild-type and alternative transcripts, and that as the SINE insertion size increases, the use of the alternative splice site increases and fewer PMEL fibers reach the melanosome (Fig. 8). In this scenario, dilute merle dogs, which have shorter oligo(dT) lengths, would produce the largest proportion of wild-type transcripts from the *Merle* allele, minimizing the effects of haploinsufficiency and resulting in less background dilution of the coat. In standard merle dogs, the longer *Merle* allele would yield more alternatively spliced transcripts and ultimately a lighter background coat color. Finally, the long *Merle* alleles of harlequin dogs would very rarely, if ever, utilize the original splice site, leading to an overwhelming production of abnormal protein.

Further study is necessary to determine the mechanism behind the lack of pigmentation observed in harlequin merles. It is possible that the sheer volume of mutant protein overwhelms the proteasome, leading to melanocyte death, as is observed in harlequin Great Danes [15]. Alternatively, some mutant PMEL might evade ubiquitination and travel to the melanosome, causing amyloidogenesis, and lead to an absence of pigmentation via the same mechanism as Dominant white chickens and Silver horses [13].

### Oligo(dT) length correlates with extent of coat variegation

Fragment analyses revealed somatic mosaicism of oligo(dT) length in standard and harlequin merles, but not cryptic or dilute merles. Standard and harlequin merle dogs possess patches of full pigmentation, while cryptic or dilute merles lack coat variegation. Taken together, these observations may suggest that a variable length of the oligo(dT) in somatic cells underlies the appearance of coat variegation. *PMEL* is expressed nearly exclusively in neural-crest derived melanocytes [4], so the variegated phenotype of standard and harlequin merles can be attributed to somatic reversions in these cells during development. We hypothesize that each individual fully pigmented spot is a clone of cells originating from a single premelanocyte in which occurred a unique somatic contraction of the oligo(dT) tract to the cryptic merle range, resulting in functional PMEL produced by both alleles.

Repetitive sequences are notoriously error prone and mosaicism is likely the byproduct of replication strand slippage, a well-studied mechanism that contributes to the evolution of DNA sequence [24–28]. During replication, if the nascent and template strands unpair on a repetitive tract, they may misalign upon reannealing, thereby causing nucleotides of the template to either be skipped (deletion) or reread (insertion) [29]. Mononucleotide repeats in particular are highly mutable, and slippage events increase in frequency as the repeat expands [24, 29, 30]. Usually replication slippage impacts just one or a few bases, although large deletions and insertions result as well [28, 31]. Here, we observed dramatic changes in oligo(dT) length. Pausing of the replication fork on the mononucleotide tract may allow the oligo(dT) to loop out, triggering repair mechanisms that cause replication slippage and consequent large contractions and expansions [28]. While we suggest that replication slippage may underlie somatic mosaicism, additional studies are required to determine the precise mechanism by which the large contractions and expansions of the oligo(dT) tract occur.

In somatic cells, we observed a clear mutational bias for oligo(dT) contraction, which is a well-documented trend in repetitive elements [27, 32, 33]. Although only 45% of standard and harlequin merle dogs had contraction events detectable in blood or buccal cells, all dogs possessed at least some spots of full pigmentation, indicating that contraction occurred in premelanocytes. We found it interesting that the blood and buccal cells obtained for fragment analyses generally reflected the overall rate of contraction in a dog, where the number and intensity of secondary, non-inherited *Merle* amplicons roughly correlate with the proportion of full pigmentation on the individual (Fig. 3). A future aim is to isolate melanocytes from skin tissues to correlate oligo(dT) length with pigmentation intensity in the fur of an individual dog.

We did not find evidence for dramatic expansion of the oligo(dT) in blood or buccal cells. Because amplification efficiency decreases as the oligo(dT) lengthens (Fig. 1d), it is possible that large expansions were present but undetectable on fragment analysis. Still, the bias for contraction provides further support that expansion has negative consequences that are unfavorable for melanocyte survival and/or proliferation.

We propose that the threshold for the mechanism behind the large contractions and deletions occurs between 75 and 78 bp, the upper and lower oligo(dT) boundaries of dilute and standard merle, respectively. Our data further illustrate that the frequency of dramatic changes is directly correlated with oligo(dT) length. Standard and harlequin merle dogs having longer oligo(dT) tracts generally exhibit more numerous and larger patches of full pigmentation, evidence for more frequent contraction events (Fig. 3). The timing of the contraction during melanocyte maturation and migration impacts patch size in that an earlier event during development will result in a larger community of cells producing normal PMEL, manifesting as a large patch or a predominantly solid coat (see Fig. 3). Ten of the 12 predominantly solid dogs in this study inherited oligo(dT) lengths in the harlequin merle range, providing further evidence for a higher frequency of slippage on longer oligo(dT) tracts.

### Germline de novo changes cause spontaneous appearance of merle varieties

Melanocytes arise from the neural crest cell population after the separation of the soma and the germline [34]; therefore, the oligo(dT) contraction events in these cells that give rise to the coat pattern are purely somatic and cannot be passed to progeny. Although not observed in our cohort, de novo mutations occurring very early in embryogenesis can rarely result in the same mutation being present in somatic and germline cells [35]. Twelve dogs in our cohort harbored de novo contractions (*n* = 5) or expansions (*n* = 7) of *Merle*, representing mutation during gametogenesis. Because we studied only individuals heterozygous for the SINE insertion, these changes in oligo(dT) length are not the product of unequal crossing over during meiotic division and are attributed to de novo mutation in the parental germline. Interestingly, nine of 12 de novo oligo(dT) length changes were inherited through the sire, a finding consistent with higher rates of mutation observed in spermatogenesis versus oogenesis [36].

## Conclusions

Our data reveal that phenotypic variability among merle dogs is caused by instability of the oligo(dT) of the *PMEL* retrotransposon. While a strong correlation exists between oligo(dT) length and phenotype, discrete allele ranges could not be defined for all of the merle varieties. This is attributed to the instability of the long mononucleotide repeat, but may also reflect genetic variation at loci involved in transcription, replication, or proteasome function.

## Additional files

Additional file 1: Study population data. Breed, phenotype, and *M* genotypes are given for all 259 dogs in our final study cohort. *Merle* amplicon and inferred oligo(dT) length in base pairs are given with corresponding signal intensity (RFU) for each dog for all peaks with an RFU value of 100 or greater. *D, H,* and *S* genotypes are given for select dogs. (XLS 66 kb)

Additional file 2: Technical replicates data. *Merle* amplicon size in base pairs is given with corresponding signal intensity (RFU) for each technical replicate. (XLS 29 kb)

## Abbreviations

aa: Amino acid
bp: Base pair
PCR: Polymerase chain reaction
RFU: Relative fluorescent unit
SINE: Short interspersed element
TMD: Transmembrane domain of PMEL
